# Racial/Ethnic and Age Group Differences in Opioid and Synthetic Opioid–Involved Overdose Deaths Among Adults Aged ≥18 Years in Metropolitan Areas — United States, 2015–2017

**DOI:** 10.15585/mmwr.mm6843a3

**Published:** 2019-11-01

**Authors:** Kumiko M. Lippold, Christopher M. Jones, Emily O’Malley Olsen, Brett P. Giroir

**Affiliations:** ^1^Office of the Assistant Secretary for Health, U.S. Department of Health and Human Services, Washington, D.C.; ^2^Oak Ridge Institute for Science and Education, Oak Ridge, Tennessee; ^3^Office of the Director, National Center for Injury Prevention and Control, CDC; ^4^Division of Overdose Prevention, National Center for Injury Prevention and Control, CDC.

Among the 47,600 opioid-involved overdose deaths in the United States in 2017, 59.8% (28,466) involved synthetic opioids (*1*). Since 2013, synthetic opioids, particularly illicitly manufactured fentanyl (IMF), including fentanyl analogs, have been fueling the U.S. overdose epidemic (*1*,*2*). Although initially mixed with heroin, IMF is increasingly being found in supplies of cocaine, methamphetamine, and counterfeit prescription pills, which increases the number of populations at risk for an opioid-involved overdose (*3*,*4*). With the proliferation of IMF, opioid-involved overdose deaths have increased among minority populations including non-Hispanic blacks (blacks) and Hispanics, groups that have historically had low opioid-involved overdose death rates (*5*). In addition, metropolitan areas have experienced sharp increases in drug and opioid-involved overdose deaths since 2013 (*6*,*7*). This study analyzed changes in overdose death rates involving any opioid and synthetic opioids among persons aged ≥18 years during 2015–2017, by age and race/ethnicity across metropolitan areas. Nearly all racial/ethnic groups and age groups experienced increases in opioid-involved and synthetic opioid–involved overdose death rates, particularly blacks aged 45–54 years (from 19.3 to 41.9 per 100,000) and 55–64 years (from 21.8 to 42.7) in large central metro areas and non-Hispanic whites (whites) aged 25–34 years (from 36.9 to 58.3) in large fringe metro areas. Comprehensive and culturally tailored interventions are needed to address the rise in drug overdose deaths in all populations, including prevention strategies that address the risk factors for substance use across each racial/ethnic group, public health messaging to increase awareness about synthetic opioids in the drug supply, expansion of naloxone distribution for overdose reversal, and increased access to medication-assisted treatment.

Drug overdose deaths were identified in the National Vital Statistics System multiple cause-of-death mortality files,* using the *International Classification of Diseases, Tenth Revision* (ICD-10), underlying cause-of-death codes X40–44 (unintentional), X60–64 (suicide), X85 (homicide), or Y10–Y14 (undetermined intent). These underlying cause-of-death codes identify deaths caused by acute toxicity from drugs rather than chronic exposure or adverse effects, including all intents. Among deaths with these underlying cause-of-death codes, the type of opioid involved in the drug overdose death is indicated by the following ICD-10 multiple cause-of-death codes: any opioid (T40.0, T40.1, T40.2, T40.3, T40.4, or T40.6) and synthetic opioids other than methadone (e.g., fentanyl, fentanyl analogs, and tramadol) (T40.4). Some deaths involved more than one type of opioid; these deaths were included in counts and rates for each subcategory. Thus, categories were not mutually exclusive.

Crude death rates per 100,000 population for overdose deaths involving any opioid and those involving synthetic opioids were examined for 2015–2017 by age group stratified by race/ethnicity within metropolitan areas (large central metro, large fringe metro, and medium/small metro). Metropolitan area was based on the 2013 urbanization classification scheme.^†^ Analyses comparing absolute and percentage changes in death rates from 2015 to 2017 used z-tests when deaths were ≥100 and nonoverlapping 95% confidence intervals based on a gamma distribution when deaths were <100.^§^ Data on synthetic opioid-involved overdose deaths by race/ethnicity and age group within nonmetropolitan areas as well as deaths among non-Hispanic American Indian/Alaska Natives, non-Hispanic Asian Americans, and persons aged <18 years were almost universally suppressed because of small numbers of deaths^¶^; thus, they were not included in the analysis.

From 2015 to 2017, death rates for drug overdoses involving any opioid and synthetic opioids increased across all racial/ethnic groups in each metropolitan area (Table 1). In large central metro areas, blacks experienced the largest absolute and percentage increases in rates of drug overdose deaths involving any opioid or synthetic opioids, with rates for deaths involving any opioid increasing 103% (from 11.8 to 24.0 per 100,000, absolute increase of 12.2), and for deaths involving synthetic opioids increasing 361% (from 3.6 to 16.6; absolute increase of 13.0). In large fringe metro areas, whites experienced the largest absolute increases rates of overdose deaths involving any opioid (from 17.8 to 26.7, absolute increase of 8.9) and those involving synthetic opioids (from 6.1 to 17.5, absolute increase of 11.4); blacks experienced the largest percentage change in drug overdose death rates involving any opioid (100%, from 7.2 to 14.4) and for overdose deaths involving synthetic opioids (332%, from 2.5 to 10.8). In medium/small metro areas, for overdose deaths involving any opioid, blacks experienced the largest percentage (82%) and absolute increase (6.0; from 7.3 to 13.3) in rates; whites had the largest absolute increase in rates of overdose deaths involving synthetic opioids (from 4.8 to 12.6, absolute increase of 7.8), and Hispanics** had the largest percentage increase in rates of drug overdose deaths involving synthetic opioids (262%, from 1.3 to 4.7).

**TABLE 1:** Opioid-involved overdose death rates and synthetic opioid–involved overdose death rates* among adults aged ≥18 years, by urbanization level,^†^ race/ethnicity,^§^and age group — National Vital Statistics System, United States, 2015–2017

Urbanization, Race/Ethnicity, Age Group (yrs)	Opioid-involved overdose deaths					Opioid-involved overdose deaths involving synthetic opioids				
	2015 no. (rate)	2016 no. (rate)	2017 no. (rate)	Absolute rate change^¶^	% Rate change^¶^	2015 no. (rate)	2016 no. (rate)	2017 no. (rate)	Absolute rate change^¶^	% Rate change^¶^
**Large central metro**										
**Black, overall**	**1,518 (11.8)**	**2,503 (19.3)**	**3,161 (24.0)**	12.2**	103^**^	**464 (3.6)**	**1,430 (11.0)**	**2,186 (16.6)**	13.0**	361**
18–24	68 (3.6)	112 (6.0)	113 (6.2)	2.6**	72**	23 (1.2)	54 (2.9)	80 (4.4)	3.2**	267**
25–34	225 (8.6)	368 (13.6)	462 (16.5)	7.9**	92**	79 (3.0)	221 (8.1)	325 (11.6)	8.6**	287**
35–44	255 (11.5)	417 (18.9)	532 (23.9)	12.4**	108**	71 (3.2)	231 (10.5)	354 (15.9)	12.7**	397**
45–54	437 (19.3)	730 (32.5)	934 (41.9)	22.6**	117**	130 (5.7)	451 (20.1)	654 (29.4)	23.7**	416**
55–64	437 (21.8)	706 (34.6)	885 (42.7)	20.9**	96**	139 (6.9)	388 (19.0)	619 (29.8)	22.9**	332**
≥65	96 (5.2)	170 (8.8)	235 (11.6)	6.4**	123**	22 (1.2)	85 (4.4)	154 (7.6)	6.4**	533**
**White, overall**	**6,636 (18.2)**	**8,251 (22.6)**	**8,989 (24.6)**	6.4**	35**	**1,743 (4.7)**	**3,633 (9.9)**	**5,038 (13.7)**	9.0**	192**
18–24	591 (16.6)	721 (20.7)	703 (20.5)	3.9**	24**	176 (4.9)	324 (9.3)	421 (12.3)	7.4**	149**
25–34	1,736 (24.8)	2,271 (32.2)	2,484 (35.2)	10.4**	42**	531 (7.6)	1,160 (16.4)	1,560 (22.1)	14.5**	191**
35–44	1,360 (24.2)	1,812 (32.4)	2,039 (36.3)	12.1**	50**	378 (6.7)	902 (16.1)	1,253 (22.3)	15.6**	232**
45–54	1,503 (24.1)	1,768 (29.0)	1,908 (32.1)	8.0**	33**	362 (5.8)	726 (11.9)	1,034 (17.4)	11.6**	199**
55–64	1,156 (18.2)	1,369 (21.5)	1,462 (23.0)	4.8**	26**	239 (3.8)	447 (7.0)	657 (10.3)	6.5**	174**
≥65	290 (3.8)	310 (3.9)	393 (4.8)	1.0**	26**	57 (0.7)	74 (0.9)	113 (1.4)	0.7**	100**
**Hispanic, overall^††^**	**1,176 (6.2)**	**1,674 (8.8)**	**1,901 (9.7)**	3.5**	57**	**238 (1.3)**	**766 (4.0)**	**1,058 (5.4)**	4.2**	350**
18–24	152 (4.9)	202 (6.5)	234 (7.6)	2.7**	55**	26 (0.8)	82 (2.7)	132 (4.3)	3.5**	438**
25–34	297 (6.8)	440 (9.9)	512 (11.2)	4.4**	65**	68 (1.5)	203 (4.6)	289 (6.3)	4.8**	320**
35–44	287 (7.2)	419 (10.5)	458 (11.3)	4.1**	57**	58 (1.5)	212 (5.3)	271 (6.7)	5.2**	347**
45–54	256 (7.8)	360 (10.8)	420 (12.3)	4.5**	58**	54 (1.7)	173 (5.2)	235 (6.9)	5.2**	306**
55–64	151 (7.0)	219 (9.8)	223 (9.5)	2.5**	36**	26 (1.2)	90 (4.0)	106 (4.5)	3.3**	275**
≥65	33 (1.7)	34 (1.7)	54 (2.5)	0.8	47	—^§§^	—	25 (1.2)	—	—
**Large fringe metro**										
**Black, overall**	**519 (7.2)**	**906 (12.3)**	**1,086 (14.4)**	7.2**	100**	**179 (2.5)**	**499 (6.8)**	**812 (10.8)**	8.3**	332**
18–24	48 (4.4)	87 (8.1)	88 (8.1)	3.7**	84**	20 (1.8)	56 (5.2)	62 (5.7)	3.9**	217**
25–34	102 (7.3)	220 (15.3)	273 (18.2)	10.9**	149**	44 (3.2)	130 (9.0)	205 (13.7)	10.5**	328**
35–44	132 (9.9)	193 (14.4)	249 (18.2)	8.3**	84**	47 (3.5)	108 (8.0)	197 (14.4)	10.9**	311**
45–54	127 (9.3)	232 (16.8)	258 (18.4)	9.1**	98**	36 (2.6)	118 (8.5)	184 (13.1)	10.5**	404**
55–64	99 (9.2)	140 (12.5)	184 (15.8)	6.6**	72**	30 (2.8)	71 (6.3)	137 (11.7)	8.9**	318**
≥65	11 (—)	34 (3.4)	34 (3.3)	—	—	—	16 (—)	27 (2.6)	—	—
**White, overall**	**7,561 (17.8)**	**10,179 (23.8)**	**11,442 (26.7)**	8.9**	50**	**2,594 (6.1)**	**5,292 (12.4)**	**7,486 (17.5)**	11.4**	187**
18–24	801 (18.5)	1,106 (25.8)	1,097 (25.9)	7.4**	40**	303 (7.0)	620 (14.5)	778 (18.4)	11.4**	163**
25–34	2,283 (36.9)	3,177 (50.9)	3,658 (58.3)	21.4**	58**	901 (14.6)	1,887 (30.3)	2,666 (42.5)	27.9**	191**
35–44	1,738 (26.9)	2,392 (37.5)	2,699 (42.4)	15.5**	58**	628 (9.7)	1,318 (20.7)	1,874 (29.4)	19.7**	203**
45–54	1,644 (20.2)	2,009 (25.1)	2,274 (29.2)	9.0**	45**	501 (6.1)	925 (11.6)	1,363 (17.5)	11.4**	187**
55–64	911 (11.4)	1,260 (15.6)	1,433 (17.6)	6.2**	54**	223 (2.8)	475 (5.9)	701 (8.6)	5.8**	207**
≥65	184 (1.9)	235 (2.4)	281 (2.8)	0.9**	47**	38 (0.4)	67 (0.7)	104 (1.0)	0.6**	150**
**Hispanic, overall^††^**	**423 (5.7)**	**674 (8.9)**	**790 (10.0)**	4.3**	75**	**123 (1.7)**	**362 (4.8)**	**531 (6.7)**	5.0**	294**
18–24	65 (5.2)	94 (7.5)	95 (7.4)	2.2**	42**	21 (1.7)	48 (3.8)	61 (4.7)	3.0**	177**
25–34	128 (7.5)	214 (12.4)	243 (13.6)	6.1**	81**	44 (2.6)	131 (7.6)	165 (9.2)	6.6**	254**
35–44	119 (7.0)	194 (11.2)	210 (11.7)	4.7**	67**	33 (1.9)	106 (6.1)	149 (8.3)	6.4**	337**
45–54	71 (5.4)	129 (9.5)	157 (11.1)	5.7**	106**	20 (1.5)	58 (4.3)	114 (8.0)	6.5**	433**
55–64	33 (4.1)	37 (4.4)	73 (8.1)	4.0**	98**	—	19 (—)	37 (4.1)	—	—
≥65	—	—	12 (—)	—	—	—	—	—	—	—
**Medium and small metro**										
**Black, overall**	**553 (7.3)**	**776 (10.1)**	**1,036 (13.3)**	6.0**	82**	**199 (2.6)**	**387 (5.0)**	**698 (8.9)**	6.3**	242**
18–24	36 (2.6)	57 (4.2)	83 (6.2)	3.6**	139**	21 (1.5)	27 (2.0)	54 (4.0)	2.5**	167**
25–34	111 (7.2)	183 (11.6)	231 (14.2)	7.0**	97**	39 (2.5)	99 (6.3)	176 (10.8)	8.3**	332**
35–44	146 (11.4)	193 (15.0)	267 (20.5)	9.1**	80**	55 (4.3)	100 (7.8)	186 (14.3)	10.0**	233**
45–54	139 (11.0)	154 (12.2)	219 (17.5)	6.5**	59**	48 (3.8)	78 (6.2)	149 (11.9)	8.1**	213**
55–64	99 (8.7)	153 (13.2)	187 (15.8)	7.1**	82**	30 (2.6)	72 (6.2)	110 (9.3)	6.7**	258**
≥65	22 (2.2)	36 (3.4)	49 (4.4)	2.2**	100**	—	11 (—)	23 (2.1)	—	—
**White, overall**	**8,794 (16.4)**	**10,530 (19.6)**	**11,767 (21.9)**	5.5**	34**	**2,547 (4.8)**	**4,449 (8.3)**	**6,803 (12.6)**	7.8**	163**
18–24	757 (11.7)	943 (14.9)	960 (15.4)	3.7**	32**	260 (4.0)	433 (6.8)	634 (10.2)	6.2**	155**
25–34	2,270 (27.7)	2,963 (35.9)	3,324 (40.2)	12.5**	45**	772 (9.4)	1,454 (17.6)	2,203 (26.6)	17.2**	183**
35–44	2,042 (26.9)	2,552 (33.9)	2,892 (38.3)	11.4**	42**	634 (8.4)	1,188 (15.8)	1,816 (24.1)	15.7**	187**
45–54	2,032 (22.6)	2,228 (25.2)	2,475 (28.7)	6.1**	27**	530 (5.9)	867 (9.8)	1,326 (15.4)	9.5**	161**
55–64	1,349 (14.0)	1,450 (14.9)	1,706 (17.5)	3.5**	25**	292 (3.0)	415 (4.3)	733 (7.5)	4.5**	150**
≥65	344 (2.7)	394 (3.0)	410 (3.1)	0.4	15	59 (0.5)	92 (0.7)	91 (0.7)	0.2**	40**
**Hispanic, overall^††^**	**709 (7.3)**	**870 (8.8)**	**1,012 (9.9)**	2.6**	36**	**127 (1.3)**	**321 (3.2)**	**485 (4.7)**	3.4**	262**
18–24	78 (4.2)	110 (5.9)	111 (5.8)	1.6**	38**	20 (1.1)	40 (2.1)	59 (3.1)	2.0**	182**
25–34	196 (8.6)	250 (10.8)	298 (12.5)	3.9**	45**	33 (1.4)	88 (3.8)	159 (6.7)	5.3**	379**
35–44	184 (9.2)	231 (11.4)	270 (12.9)	3.7**	40**	37 (1.9)	103 (5.1)	138 (6.6)	4.7**	247**
45–54	159 (10.2)	166 (10.4)	199 (12.1)	1.9	19	29 (1.9)	57 (3.6)	87 (5.3)	3.4**	179**
55–64	77 (7.3)	93 (8.4)	117 (10.1)	2.8**	39**	—	29 (2.6)	38 (3.3)	—	—
≥65	15 (—)	20 (2.0)	17 (—)	—	—	—	—	—	—	—

* Deaths were classified using the *International Classification of Diseases, Tenth Revision* (ICD-10). Opioid-involved overdose deaths were identified using underlying cause-of-death codes X40–44, X60–64, X85, and Y10–14. Among deaths with overdose as the underlying cause, the type of drug involved in the overdose death was indicated by the following ICD-10 multiple cause-of-death codes: any opioid (T40.0, T40.1, T40.2, T40.3, T40.4, or T40.6) and synthetic opioids other than methadone (T40.4). Totals for deaths by category might involve more than one drug other than synthetic opioids. Rates displayed are age-specific crude rates per 100,000 persons.

^†^ Based on the 2013 urbanization classification (https://www.cdc.gov/nchs/data_access/urban_rural.htm). *Large central metro:* counties in metropolitan statistical areas (MSAs) of ≥1 million population that 1) contain the entire population of the largest principal city of the MSA, or 2) have their entire population contained in the largest principal city of the MSA, or 3) contain at least 250,000 inhabitants of any principal city of the MSA. *Large fringe metro:* counties in the MSAs of ≥1 million population that did not qualify as large central metro counties. *Medium metro:* counties in MSAs of populations of 250,000–999,999. *Small metro:* counties in MSAs of populations <250,000. Because of low numbers of deaths and rate suppression for key populations, micropolitan areas (nonmetropolitan counties) and noncore areas (counties that did not qualify as micropolitan) were not included in this analysis.

^§^ Blacks and whites are non-Hispanic; Hispanic persons can be of any race.

^¶^ Absolute rate change is the difference between the 2015 and 2017 rates. Percent change in rate is calculated as the absolute rate change divided by the 2015 rate, multiplied by 100. Statistical significance was determined using nonoverlapping 95% confidence intervals (CIs) based on the gamma method if the number of deaths was <100 in 2015 and 2017, and z-tests were used if the number of deaths was ≥100 in 2015 and 2017. Percent changes were rounded to the nearest whole number. The method of comparing CIs is a conservative method for statistical significance, and caution should be used when interpreting a nonsignificant difference when the lower and upper bounds being compared only slightly overlap.

** p<0.05 using z-tests when deaths were ≥100 or when deaths were <100; nonoverlapping 95% CIs based on a gamma distribution.

^††^ Data for Hispanic origin should be interpreted with caution; studies comparing Hispanic origin on death certificates and on census surveys have indicated that reporting on Hispanic ethnicity is inconsistent. https://www.cdc.gov/nchs/data/series/sr_02/sr02_172.pdf.

^§§^ Dashes indicate that result is suppressed because <10 deaths, and rates based on <20 deaths are considered unreliable. Absolute and percent changes in rates cannot be calculated for these values.

Examining death rates for drug overdose deaths involving any opioid or synthetic opioids by racial/ethnic age groups in large central metro areas found that the highest drug overdose death rates involving any opioid (42.7) and synthetic opioids (29.8) in 2017 were among blacks aged 55–64 years (Table 1). From 2015 to 2017, blacks aged 45–54 years in large central metro areas experienced the largest absolute increase in death rates involving any opioid (from 19.3 to 41.9, absolute increase of 22.6) and synthetic opioids (from 5.7 to 29.4, absolute increase of 23.7), and blacks aged ≥65 years in these areas had the largest percentage increases in rates of drug overdose deaths involving any opioid (123%; from 5.2 to 11.6) and synthetic opioids (533%; from 1.2 to 7.6).

Among racial/ethnic age groups in large fringe metro areas, in 2017, the highest rates of drug overdose deaths involving any opioid (58.3) and synthetic opioids (42.5) were in whites aged 25–34 years (Table 1); this group also experienced the largest absolute increases in death rates involving any opioid (from 36.9 to 58.3; absolute increase of 21.4) and synthetic opioids (from 14.6 to 42.5; absolute increase of 27.9) in these areas from 2015 to 2017. The largest percentage increase in rates of drug overdose deaths involving any opioid in large fringe metro areas from 2015 to 2017 occurred among blacks aged 25–34 years (149%; from 7.3 to 18.2), and the largest percentage increase in overdose death rates involving synthetic opioids was in Hispanics aged 45–54 years (433%; from 1.5 to 8.0).

Among racial/ethnic age groups in medium/small metro areas, in 2017, the highest rates of drug overdose deaths involving any opioid or synthetic opioids were in whites aged 25–34 years (40.2 and 26.6, respectively). This group also experienced the largest absolute increases in drug overdose death rates involving any opioid (from 27.7 to 40.2, absolute increase of 12.5) and synthetic opioids (from 9.4 to 26.6, absolute increase of 17.2) in these areas from 2015 to 2017 (Table 1). From 2015 to 2017, blacks aged 18–24 years experienced the largest percentage increase in opioid-involved overdose death rates (139%; from 2.6 to 6.2); the largest percentage increase in synthetic opioid–involved overdose death rates (379%; from 1.4 to 6.7) occurred among Hispanics aged 25–34 years.

The percentage of all opioid-involved overdose deaths involving synthetic opioids increased from 2015 to 2017 across all racial/ethnic age groups in each metropolitan area category (Table 2). By 2017, the greatest level of synthetic opioid involvement in opioid-involved overdose deaths was among blacks in all metro areas and ranged from 67.4% in medium/small metro areas to 74.8% in large fringe metro areas. Among whites, the percentage of opioid-involved overdose deaths involving synthetic opioids ranged from 56.0% in large central metro areas to 65.4% in large fringe metro areas. Among Hispanics, the percentage of opioid-involved overdose deaths involving synthetic opioids ranged from 47.9% in medium/small metro areas to 67.2% in large fringe metro areas.

**TABLE 2:** Percentage of opioid-involved overdose deaths* involving synthetic opioids among adults aged ≥18 years, by urbanization level, age group, and race/ethnicity, — National Vital Statistics System, United States, 2015–2017

Urbanization level^†^	Age group (yrs)	Race/Ethnicity^§,¶^	Year, %			
			2015	2016	2017	% Increase, 2015–2017**^,††^
Large central metro	All	Black	30.6	57.1	69.2	126
		White	26.1	44.0	56.0	115
		Hispanic	20.2	45.8	55.7	175
	18–24	Black	33.8	48.2	70.8	109
		White	29.8	44.9	59.9	101
		Hispanic	17.1	40.6	56.4	230
	25–34	Black	35.1	60.1	70.3	100
		White	30.6	51.1	62.8	105
		Hispanic	22.9	46.1	56.4	147
	35–44	Black	27.8	55.4	66.5	139
		White	27.8	49.8	61.5	121
		Hispanic	20.2	50.6	59.2	193
	45–54	Black	29.7	61.8	70.0	135
		White	24.1	41.1	54.2	125
		Hispanic	21.1	48.1	56.0	165
	55–64	Black	31.8	55.0	69.9	120
		White	20.7	32.7	44.9	117
		Hispanic	17.2	41.1	47.5	176
	≥65	Black	22.9	50.0	65.5	186
		White	19.7	23.9	28.8	46
		Hispanic	—^§§^	—	46.3	—
Large fringe metro	All	Black	34.5	55.1	74.8	117
		White	34.3	52.0	65.4	91
		Hispanic	29.1	53.7	67.2	131
	18–24	Black	41.7	64.4	70.5	69
		White	37.8	56.1	70.9	88
		Hispanic	32.3	51.1	64.2	99
	25–34	Black	43.1	59.1	75.1	74
		White	39.5	59.4	72.9	85
		Hispanic	34.4	61.2	67.9	98
	35–44	Black	35.6	56.0	79.1	122
		White	36.1	55.1	69.4	92
		Hispanic	27.7	54.6	71.0	156
	45–54	Black	28.3	50.9	71.3	152
		White	30.5	46.0	59.9	97
		Hispanic	28.2	45.0	72.6	158
	55–64	Black	30.3	50.7	74.5	146
		White	24.5	37.7	48.9	100
		Hispanic	—	—	50.7	—
	≥65	Black	—	—	79.4	—
		White	20.7	28.5	37.0	79
		Hispanic	—	—	—	—
Medium and small metro	All	Black	36.0	49.9	67.4	87
		White	29.0	42.3	57.8	100
		Hispanic	17.9	36.9	47.9	168
	18–24	Black	58.3	47.4	65.1	12
		White	34.3	45.9	66.0	92
		Hispanic	25.6	36.4	53.2	108
	25–34	Black	35.1	54.1	76.2	117
		White	34.0	49.1	66.3	95
		Hispanic	16.8	35.2	53.4	217
	35–44	Black	37.7	51.8	69.7	85
		White	31.0	46.6	62.8	102
		Hispanic	20.1	44.6	51.1	154
	45–54	Black	34.5	50.6	68.0	97
		White	26.1	38.9	53.6	106
		Hispanic	18.2	34.3	43.7	140
	55–64	Black	30.3	47.1	58.8	94
		White	21.6	28.6	43.0	99
		Hispanic	—	31.2	32.5	—
	≥65	Black	—	—	46.9	—
		White	17.2	23.4	22.2	29
		Hispanic	—	—	—	—

* Deaths were classified using the *International Classification of Diseases, Tenth Revision* (ICD-10). Opioid-involved overdose deaths were identified using underlying cause-of-death codes X40–44, X60–64, X85, and Y10–14. Among deaths with overdose as the underlying cause, the type of drug involved in the overdose death was indicated by the following ICD-10 multiple cause-of-death codes: any opioid (T40.0, T40.1, T40.2, T40.3, T40.4, or T40.6) and synthetic opioids other than methadone (T40.4). Totals for deaths by category might involve more than one drug other than synthetic opioids. The percentage of opioid-involved overdose deaths involving synthetic opioids was calculated by dividing the number of opioid-involved overdose deaths involving synthetic opioids by the number of opioid-involved overdose deaths, then multiplying by 100.

^†^ Based on the 2013 urbanization classification (https://www.cdc.gov/nchs/data_access/urban_rural.htm). *Large central metro:* counties in metropolitan statistical areas (MSAs) of ≥1 million population that 1) contain the entire population of the largest principal city of the MSA, or 2) have their entire population contained in the largest principal city of the MSA, or 3) contain at least 250,000 inhabitants of any principal city of the MSA. *Large fringe metro:* counties in the MSAs of ≥1 million population that did not qualify as large central metro counties. *Medium metro:* counties in MSAs of populations of 250,000–999,999. *Small metro:* counties in MSAs of populations <250,000. Because of low numbers of deaths and rate suppression for key populations, micropolitan areas (nonmetropolitan counties) and noncore areas (counties that did not qualify as micropolitan) were not included in this analysis.

^§^ Blacks and whites were non-Hispanic; Hispanics could be of any race.

^¶^ Data for Hispanic origin should be interpreted with caution; studies comparing Hispanic origin on death certificates and on census surveys have indicated that reporting on Hispanic ethnicity is inconsistent. https://www.cdc.gov/nchs/data/series/sr_02/sr02_172.pdf.

** Percentage increase in opioid-involved overdose deaths involving synthetic opioids was calculated by subtracting the percentage of deaths that involved synthetic opioids in 2017 from the percentage of deaths involving synthetic opioids in 2015, dividing this value by the percentage of deaths involving synthetic opioids in 2015, and then multiplying by 100.

^††^ Total percent changes were rounded to the nearest whole number.

^§§^ Dashes indicate that percent change in synthetic opioid involvement in opioid-involved overdose deaths could not be calculated because of unreliable rates or suppression.

## Discussion

Synthetic opioids are driving the recent increases in opioid-involved overdose deaths in the United States. Previous research has found that synthetic opioids were involved in nearly 60% of opioid-involved overdose deaths in the United States in 2017 (*1*); this study examines the variation in synthetic opioid involvement in these deaths among racial/ethnic age groups across different metropolitan areas. For example, in large central metro areas, among persons aged 45–54 years, synthetic opioids were involved in 70.0% of all opioid-involved overdose deaths among blacks, 54.2% among whites, and 56.0% among Hispanics. These findings underscore the changing demographics and populations affected by the opioid overdose epidemic as the illicit drug supply continues to evolve.

Consistent with these findings, a recent report by the New York City Department of Health and Mental Hygiene (*8*) identified high rates of drug overdoses in 2017 involving heroin or fentanyl among middle-aged and older-aged blacks and Hispanics in a large metropolitan area infiltrated by IMF in recent years; these rates have largely eclipsed those among whites of the same age (*9*). The distinct age distributions of opioid-involved overdose deaths between the racial/ethnic age groups and different metropolitan areas highlight the heterogeneity that exists among persons who use drugs, illicit drug markets, and risk factors for overdose. Differences in opioid prescribing rates, underlying rates of opioid and other substance use disorders, access to substance use disorder treatment, and the proliferation of IMF in the illicit drug supply might all underlie the unique patterns of opioid-involved overdose deaths observed in this study. Thus, additional efforts are needed to develop and implement prevention, treatment, and response strategies that are tailored to diverse racial/ethnic and age groups within specific community contexts. In addition, more research is needed to explore the underlying drivers of differing overdose risk among racial/ethnic age groups across metropolitan areas.

The findings in this report are subject to at least four limitations. First, numbers and rates of deaths involving specific drugs might be affected by factors related to death investigations, such as the substances tested for or the circumstances under which these tests are performed. Second, changes in fentanyl or other synthetic opioid testing and reporting as well as the percentage of deaths with specific drugs listed on the death certificate have changed over the study period and might have contributed to the observed increases in opioid- and synthetic opioid–involved overdose deaths.^††^ Third, potential racial or ethnic misclassification might lead to underestimates or overestimates for certain groups. Finally, because of small numbers of synthetic opioid–involved overdose deaths among certain racial/ethnic groups, persons aged <18 years, and in nonmetropolitan areas, data on these populations were not included in this report. Thus, exploration of how synthetic opioids are affecting these populations is beyond the scope of this report.

The changing patterns of the opioid overdose epidemic necessitate a rapid, culturally tailored and multifaceted public health response that appropriately targets and incorporates the needs of evolving populations at risk, including minority populations that historically have been regarded as having low opioid-involved overdose death rates. Curbing the opioid overdose epidemic requires collaborations across all sectors of government, law enforcement, public health, and communities. This study emphasizes the importance of data-informed approaches to addressing the evolving needs of communities and highlights the need for timely data that can be used to effectively guide public health responses. Prevention and response strategies include public health messaging campaigns to increase awareness about illicit synthetic opioids in the drug supply, naloxone distribution that targets both persons who knowingly use opioids and those who might be exposed to opioids through contamination of other illicit drugs, the expansion of and equitable access to medication-assisted treatment for opioid use disorder, evidence-based treatment for other substance use disorders, and recovery support services for persons with substance use disorders. Importantly, cultural, language, and structural barriers that minority populations might face should be considered as these interventions are being developed and implemented.

**Summary**

**What is already known about this topic?**

Opioid-involved overdose death rates in the United States differ by demographic and geographic characteristics. Illicitly manufactured fentanyl and fentanyl analogs have fueled recent increases in opioid-involved overdose deaths. In 2017, synthetic opioids were involved in nearly 60% of opioid-involved overdose deaths; however, the level of involvement by racial/ethnic age groups in metropolitan areas has not been explored.

**What is added by this report?**

From 2015 to 2017, nearly all racial/ethnic groups and age groups experienced significant increases in opioid-involved and synthetic opioid–involved overdose death rates, particularly blacks aged 45–54 years (from 19.3 to 41.9 per 100,000) and 55–64 years (from 21.8 to 42.7) in large central metro areas. The increased involvement of synthetic opioids in overdose deaths is changing the demographics of the opioid overdose epidemic.

**What are the implications for public health practice?**

Culturally competent interventions are needed to target populations at risk; these interventions include increasing awareness about synthetic opioids in the drug supply and expanding evidence-based interventions, such as naloxone distribution and medication-assisted treatment.

## Footnotes

* https://www.cdc.gov/nchs/nvss/mortality_public_use_data.htm.

† Based on 2013 urbanization classification (https://www.cdc.gov/nchs/data_access/urban_rural.htm). *Large central metro:* counties in metropolitan statistical areas (MSAs) of ≥1 million population that 1) contain the entire population of the largest principal city of the MSA, or 2) have their entire population contained in the largest principal city of the MSA, or 3) contain at least 250,000 inhabitants of any principal city of the MSA (e.g., District of Columbia and New York County, New York). *Large fringe metro:* counties in the MSAs of ≥1 million population that did not qualify as large central metro counties (e.g., Baltimore County, Maryland, and Austin County, Texas). *Medium metro:* counties in MSAs of populations of 250,000–999,999 (e.g., Durham County, North Carolina). *Small metro:* counties in MSAs of populations <250,000 (e.g., Montgomery County, Virginia). For this study, medium and small metros are combined (medium/small).

§ https://www.cdc.gov/nchs/data/nvsr/nvsr63/nvsr63_03.pdf.

¶ Death counts are suppressed when the result is fewer than 10 death because of confidentiality constraints that aid in the protection of personal privacy and prevent identification.

** Data for Hispanic origin should be interpreted with caution; studies comparing Hispanic origin on death certificates and on census surveys have indicated that reporting on Hispanic ethnicity is inconsistent. https://www.cdc.gov/nchs/data/series/sr_02/sr02_172.pdf.

†† In 2016 and 2017, 15 and 12% of death certificates, respectively, did not include mention of the type of specific drug involved in the overdose death. The percentage of death certificates that specified at least one drug varied between states and ranged from 54.7% to 99.7% in 2017.
